# Monolithic Wafer Scale Integration of Silicon Nanoribbon Sensors with CMOS for Lab-on-Chip Application

**DOI:** 10.3390/mi9110544

**Published:** 2018-10-25

**Authors:** Ganesh Jayakumar, Per-Erik Hellström, Mikael Östling

**Affiliations:** KTH Royal Institute of Technology, Department of Electronics, School of Electrical Engineering and Computer Science, 16440 Kista, Sweden; pereh@kth.se (P.-E.H.); ostling@kth.se (M.Ö.)

**Keywords:** silicon ribbon pixel, silicon ribbon biosensor, lab-on-chip, SiRi CMOS integration, selective multiplexed detection, SiRi frontgate mode, SiRi backgate mode

## Abstract

Silicon ribbons (SiRi) have been well-established as highly sensitive transducers for biosensing applications thanks to their high surface to volume ratio. However, selective and multiplexed detection of biomarkers remains a challenge. Further, very few attempts have been made to integrate SiRi with complementary-metal-oxide-semiconductor (CMOS) circuits to form a complete lab-on-chip (LOC). Integration of SiRi with CMOS will facilitate real time detection of the output signal and provide a compact small sized LOC. Here, we propose a novel pixel based SiRi device monolithically integrated with CMOS field-effect-transistors (FET) for real-time selective multiplexed detection. The SiRi pixels are fabricated on a silicon-on-insulator wafer using a top-down method. Each pixel houses a control FET, fluid-gate (FG) and SiRi sensor. The pixel is controlled by simultaneously applying frontgate (V_G_) and backgate voltage (V_BG_). The liquid potential can be monitored using the FG. We report the transfer characteristics (I_D_-V_G_) of N- and P-type SiRi pixels. Further, the I_D_-V_G_ characteristics of the SiRis are studied at different V_BG_. The application of V_BG_ to turn ON the SiRi modulates the subthreshold slope (SS) and threshold voltage (V_TH_) of the control FET. Particularly, N-type pixels cannot be turned OFF due to the control NFET operating in the strong inversion regime. This is due to large V_BG_ (≥25 V) application to turn ON the SiRi sensor. Conversely, the P-type SiRi sensors do not require large V_BG_ to switch ON. Thus, P-type pixels exhibit excellent I_ON_/I_OFF_ ≥ 10^6^, SS of 70–80 mV/dec and V_TH_ of 0.5 V. These promising results will empower the large-scale cost-efficient production of SiRi based LOC sensors.

## 1. Introduction

Silicon ribbon (SiRi) field-effect-transistors (FETs) have been widely recognized as efficient standalone prostate specific antigen (PSA) cancer marker, DNA, virus and pH sensors [1,2,3,4,5,6,7,8,9]. The small size, high surface to volume ratio, electrical read-out and a dimension that is comparable to the target of interest make SiRi-FETs an excellent contender for label-free pH and bio detection [1,2,3,4,5,6,7,8,9]. Table 1 summarizes some of the most important works employing SiRi sensor for the detection of pH or bio targets.

**Figure 1:**
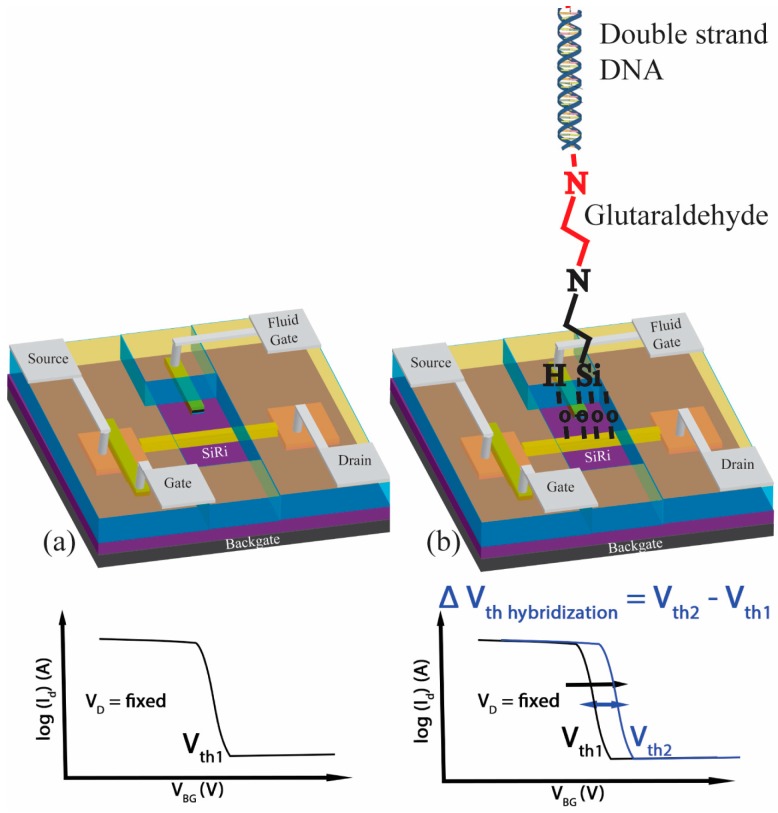
A schematic depicting the working mechanism of a silicon ribbon (SiRi) sensor. (**a**) The threshold voltage (V_th1_) of the SiRi sensor is measured before bio molecule addition. (**b**) The target double strand DNA molecule is added on the surface where it undergoes hybridization process. As a result, the surface charge on the SiRi changes. The threshold voltage of the SiRi is measured again (V_th2_). By noting the change in the threshold voltage (V_th2_ − V_th1_), the added charge on the surface can be estimated.

The sensing mechanism of the SiRi-FET is based on the principle of detection of surface charge (Figure 1). Initially, the current flowing through the SiRi is measured without any DNA hybridization and its threshold voltage is noted (V_th1_) (Figure 1a). Later, the surface of the SiRi is functionalized with the receptors and target of interest such as a double stranded DNA. Then, the current flowing through the SiRi is re-measured. The addition of DNA molecule on the surface and the resulting hybridization process causes a change in the surface charge density (Q_hybrid_). As a result, it changes the threshold voltage of the SiRi (V_th2_) (Figure 1b). By noting the difference in the threshold voltages (ΔV_th hybridization_ = V_th2_ − V_th1_) before and after DNA addition, the amount of additional charge (N_hybrid_) resulting from the target molecule of interest can be estimated using Equation (1) [10].

N_hybrid_ = Q_hybrid_/q = −∆V_th_ (ε_0_ε_r_)/(qt_ox_) (1)

In particular, the SiRi-FETs described in this work can be functionalized using the process shown by Nguyen et al. [5] and Jayakumar et al. [11]. The first step in the DNA probe covalent grafting process is silanization. In this step, the sensor surface is functionalized with single strand DNA using an organosilane such as (3-Aminopropyl)triethoxysilane (APTES) [5]. Later, the sensor can be used for the hybridization detection of target DNA that is complementary to the probe DNA [11]. At the same step, to verify the selectivity of the sensor, its surface is exposed to non-complementary DNA and saline buffer solution that is free of complementary DNA [11]. A well-behaved SiRi sensor is expected to not respond to the non-complementary DNA or the salt crystallization after drying and only exhibit V_TH_ shifts because of the target complementary DNA [11]. Indeed, the sensing mechanism employed by works listed in Table 1 relies on the conductance changes in the SiRi channel. However, as shown in our previous work, during the electrical characterization of the sensor (after silanization, grafting and hybridization steps), the current ratio (I_ON_/I_OFF_) and subthreshold slope (SS) of the SiRi-FET almost remain constant. The transfer characteristics of the sensor only translate as shift in the threshold voltage [11] (Figure 1). This method of detection ensures that there is no fluctuation of the drain current during sensing and the field effect electrostatic coupling occurs between the charges on the SiRi surface and the channel [11]. The biodetection efficiency of such a DNA hybridization detection process is characterized by the sensitivity parameter, which is defined as the threshold voltage shift (V_th2_ − V_th1_) after hybridization [11]. 

The attractive benefits of SiRi sensors can be further extended to selective target detection, multi-target detection, and synchronous real-time read-out by integrating them with complementary metal oxide semiconductor (CMOS) field-effect transistors (FETs) and circuits [12]. Furthermore, the integration of SiRi sensors with CMOS read-out circuits establishes a platform for the realization of a complete lab-on-chip (LOC) sensor. However, as can be seen in Table 1, even though many researchers have successfully used the standalone SiRi sensor for detection of pH and bio-targets, none of the works address the monolithic integration of SiRi with CMOS. The goal of this paper is to establish one such platform. We achieve the SiRi CMOS integration by employing a pixel-based LOC architecture. The pixel-based SiRi LOC design is akin to the CMOS image sensor pixel design [13]. 

**Figure 2:**
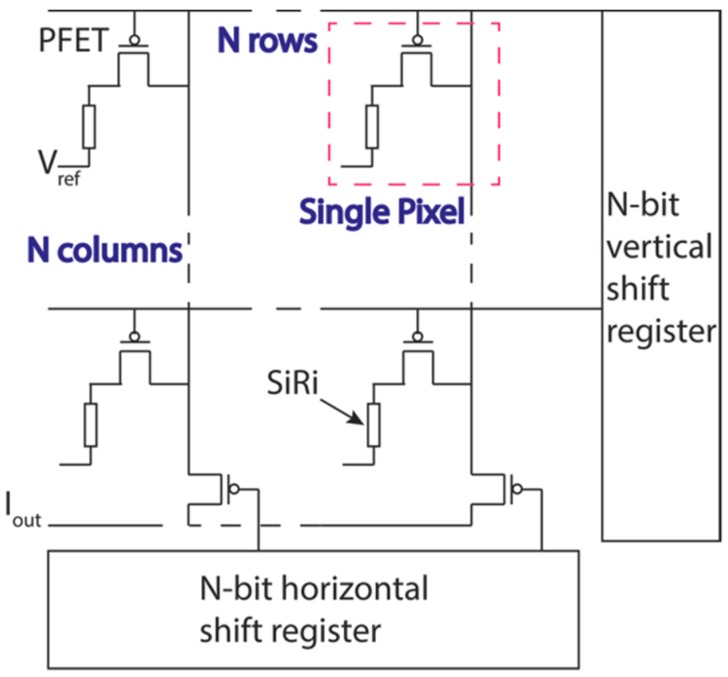
Circuit diagram of SiRi pixel based lab-on-chip (LOC). Each SiRi is connected to an on-chip fluid gate and a control transistor forming a SiRi pixel [14]. “N × N” matrix of such pixels can be addressed using N-bit vertical and horizontal shift register circuits, respectively.

Figure 2 shows the proposed scalable sequential read-out scheme to integrate SiRi sensors with CMOS. The details of the circuits and the layout are described elsewhere [14]. Here, we highlight the salient features relevant to SiRi integration. In the pixel based integration scheme, each SiRi is connected to an on-chip fluid gate and a control transistor forming a SiRi pixel [14]. “N × N” matrix of such pixels can be addressed using N-bit vertical and horizontal shift register circuits, respectively. In this design, the transistor is configured as a switch to particularly control a specific row or column. Especially, the on-chip fluid gate is integrated in the design to monitor the potential in the liquid environment. The advantage of such a design is that the output current of the pixel array could further be monolithically connected to amplifiers for signal amplitude magnification, band pass filters for reducing the noise or allowing only a certain range of the signal and other read-out circuits such as a trans-impedance amplifiers to convert current to voltage output [12].

To avail such desirable benefits of CMOS integration, the manufacturing scheme employed for the realization of SiRi pixel sensor also has to be CMOS compatible. Traditionally, there are two methods to manufacture SiRi sensors: (1) bottom-up (BU); and (2) top-down (TD) [15,16,17]. In the BU method, the SiRi sensors are realized by using molecular pre-cursors. For example a silicon nanonet field effect transistor (SiNN-FET) can be manufactured on a bulk Si wafer using the BU method [5,18]. On the contrary, in the TD method, SiRi sensors are commonly realized on a silicon-on-insulator (SOI) substrate using the well-established lithography and patterning techniques [2,3,4,6,7,8,9,19]. It is particularly challenging to transfer the bottom-up grown nanonets on to a CMOS wafer and continue processing due to the tangling of the grown wires, difficulty in alignment and manual errors [20]. Further, in the bottom-up method, the probability of introducing contaminants on to the wafer is very high [18,20]. It also demands the usage of specialized alignment methods that tend to be time consuming [18,20]. In comparison, the top down method is CMOS compatible and allows for the low-cost co-integration of SiRi sensors with metal-oxide-semiconductor field-effect transistors (MOSFETs) and circuits [14,15,16,17]. 

Therefore, in this article, we utilize the fully CMOS compatible top-down method to fabricate the SiRi pixel sensors on wafer scale. Then, we show the first electrical results of the pixel design for both N- and P-type SiRi pixels. Single SiRi sensors of dimensions width W= 1 μm and length L = 1 μm were connected to N- and P-type CMOS transistors (W = 4 μm and L = 1 μm) to fully exploit the monolithic integration of SiRi with CMOS transistors. The SiRi pixel can be operated in two different modes: (a) the frontgate mode; and (b) the backgate mode. In the frontgate mode, the pixel is turned ON or OFF by applying a frontgate voltage to the N- and P-type transistors, while the backgate bias is fixed. In the backgate mode, the pixel is turned ON or OFF by applying a backgate voltage to the substrate, while the frontgate bias of the N- and P-type transistors is fixed. Both modes of operation validate the concept of establishing control of individual SiRi pixel modules in a matrix of sensors. Indeed, these promising results will empower the large-scale cost efficient production of compact label-free SiRi based lab-on-chip (LOC) sensors.

## 2. Materials and Methods

**Figure 3:**
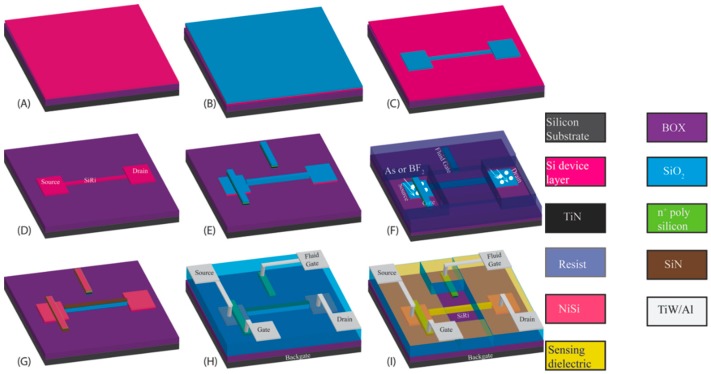
Schematic of the process steps employed in the manufacturing of the SiRi pixel sensor: (**A**) SOI wafer starting substrate; (**B**) deposition of 40 nm SiO_2_ hard mask; (**C**) lithography and RIE of SiO_2_ hard mask to define the contact pads of the control transistor and SiRi channel; (**D**) reactive ion etching (RIE) of the crystalline silicon (c-Si) device layer using SiO_2_ mask and transfer of the pattern to c-Si device layer; (**E**) thermal gate-oxide growth, TiN gate metal and n^+^ polysilicon deposition and patterning to define the frontgate of the transistor and fluid gate of the SiRi pixel; (**F**) As or BF_2_ ion-implantation step using resist mask to form N-metal-oxide-semiconductor (NMOS) and P-metal-oxide-semiconductor (PMOS) pixels respectively; (**G**) after dopant activation and SiO_2_/SiN spacer formation along the gate sidewall, NiSi ohmic contact formation; (**H**) contact hole definition and TiW/Al metal patterning; and (**I**) etching pathways to access the SiRi test site using lithography mask and RIE etching. Later, a 10 nm atomic layer deposition (ALD) SiO_2_ passivation oxide that also behaves as sensing dielectric is deposited.

The important steps in the fabrication of the SiRi pixel sensor are shown in Figure 3. The SiRi pixel sensors were fabricated on boron doped (1 × 10^15^ cm^−3^) 4” (100 mm) SOI wafer. A 145 nm buried oxide layer (BOX) separates the top 55 nm crystalline silicon (c-Si) device layer from the substrate. The c-Si device layer was thermally oxidized at 1000 °C and thinned down to 20 nm (Figure 3A).

Then, 40 nm SiO_2_ hard mask was deposited by plasma enhanced chemical vapor deposition (PECVD) at 400 °C (Figure 3B). The SiRi pattern and active region was defined on the SiO_2_ surface using conventional I-line lithography. Using a resist mask the SiO_2_ was selectively etched towards c-Si in CHF_3_/CF_4_/O_2_ plasma (Figure 3C). Then, the 20 nm c-Si device layer was etched using SiO_2_ as mask in Cl_2_/HBr/O_2_ plasma (Figure 3D). The final dimension of the SiRi sensor is 1 μm × 1 μm × 20 nm (L × W × H). Next, a diluted HF spray etch was employed to strip the SiO_2_ on top of source/drain pads which recessed the BOX to 140 nm. Later, the active region of the SiRi pixel was thermally oxidized at 800 °C to form 5 nm SiO_2_. This 5 nm SiO_2_ forms the gate dielectric material for the control transistor in the SiRi pixel. This step was followed by 10 nm physical vapor deposition (PVD) of TiN gate metal, low-pressure chemical vapor deposition (LPCVD) of 100 nm n^+^ polysilicon and PECVD deposition of 40 nm SiO_2_ hard mask.

The transistor gate and on-chip fluid gate were patterned using I-line lithography and without breaking vacuum in the reactive ion etching (RIE) chamber the n^+^ poly silicon/ TiN stack was selectively etched towards underlying c-Si active region using Cl_2_/HBr/O_2_ and BCl_3_/Cl_2_ plasma, respectively (Figure 3E). Next, using a resist mask that covers the SiRi region, the active regions of the SiRi pixel was implanted. To form N-type SiRi pixels, the source/drain regions was doped with As^+^, whereas, to form P-type SiRi pixels, the source/drain regions were doped with BF_2_ (Figure 3F).

In the cases of N and P SiRi pixels, the dose of 1 × 10^15^ cm^−3^ and energy 10 keV was optimized based on iterations in semiconductor simulation software SRIM (2013 version) developed by James F. Ziegler, USA. The 40 nm SiO_2_ gate etch mask and thin layer of SiO_2_ gate dielectric material was preserved during the implantation to avoid surface damage and 7° tilt angle was opted to avoid channeling. After resist stripping, a thin layer of 10 nm SiO_2_ was deposited by atomic layer deposition (ALD) followed by deposition of an additional 60 nm SiN. The SiO_2_/SiN stack was patterned using I-line lithography and etched everywhere except on-top of the SiRi. This SiO_2_/SiN stack is later used as a mask during the salicide process step to prevent silicidation of the SiRi. 

After the SiO_2_/SiN patterning, a rapid thermal annealing (RTA) step followed at 1000 °C for 10 s to activate the dopants. Next, ~40 nm gate SiO_2_ mask and ~5 nm thermal oxide/10 nm SiO_2_ lying on top of the source/drain region is cleared using diluted HF spray etch. A 5 nm Ni was deposited using PVD and annealed at 450 °C for 30 s to form low resistance ohmic source/drain and gate contacts (Figure 3G). The unreacted Ni was stripped in H_2_SO_4_:H_2_O_2_ (3:1) mixture. Then, 400 nm PECVD SiO_2_ was deposited, patterned using I-line lithography and RIE was done in CHF_3_/CF_4_/O_2_ plasma to define contact hole for the SiRi pixel. After metallization using PVD 100 nm TiW lining layer and 500 nm Al layer, patterning and SF_6_/BCl_3_/Cl_2_ RIE etching (Figure 3H), 100 nm PECVD passivation oxide layer was deposited to protect the metal lines and the transistor.

**Figure 4:**
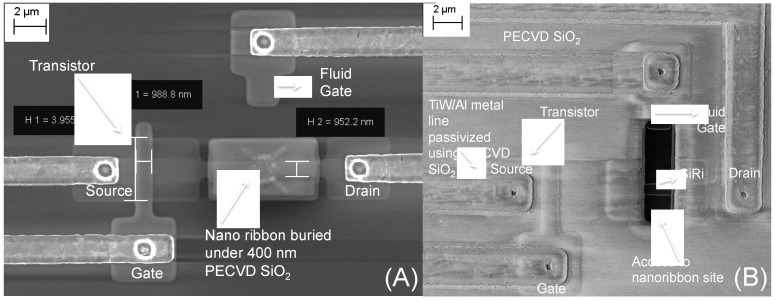
Top view scanning electron microscope (SEM) image of a single SiRi pixel showing the control FET, fluid gate and SiRi sensor: (**A**) before opening access to the SiRi test site; and (**B**) after opening access to the SiRi test site.

The top-view scanning electron microscope (SEM) image of a typical single SiRi pixel device after the metallization step but prior to opening access to SiRi test site is shown in Figure 4A. At this stage, the 500 nm SiO_2_ is still lying on-top of the SiRi along with the 10 nm SiO_2_/60 nm SiN stack that was deposited to mask silicidation process. The sensing area of the SiRi channel was exposed by using a resist mask, I-line lithography step and selective RIE of ~570 nm dielectric in CHF_3_/CF_4_/O_2_ plasma. Then, 10 nm ALD SiO_2_ was deposited at 350 °C on top of the SiRi sensor (Figure 3I).

The ALD SiO_2_ will behave as the passivation layer on top of the sensor as well as act as the sensing dielectric during the bio functionalization experiments. Figure 4B shows the top-view SEM image of a SiRi pixel after opening access to SiRi test site. To probe the SiRi pixels, the 100 nm passivation oxide lying on-top of the bondpads was etched using CHF_3_/CF_4_/O_2_ plasma. Finally, a forming gas anneal was done at 400 °C and marks the completion of the entire fabrication process.

## 3. Electrical Characterization

**Figure 5:**
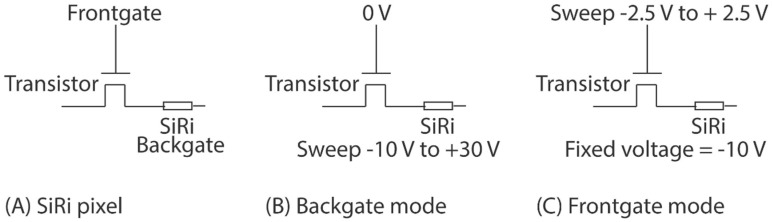
(**A**) The test setup for electrical evaluation of the SiRi pixel sensors in (**B**) backgate mode where frontgate of the control transistor is fixed at 0 V while the backgate of the SiRi is swept from −10 to +30 V and (**C**) frontgate mode where the transistor frontgate is swept from −2.5 to 2.5 V and SiRi is biased at fixed voltage where it is always ON (−10 V for P-type SiRi).

The setup shown in Figure 3I was used for the electrical evaluation of the SiRi pixel sensors prior to functionalization. A Cascade 12,000 semi-automatic wafer prober (Cascade Microtech, Beaverton, OR, USA) that was externally connected to a Keithley 4200-SCS parameter analyzer (Tektronix, Inc., Beaverton, OR, USA) was used to perform direct current (DC) electrical measurements. It also facilitated full-scale wafer mapping. In the pixel module, the source terminal of the transistor is connected to the column selection line while the drain terminal of the transistor is connected to the SiRi. The SiRi pixel can be operated in two modes—the backgate mode (Figure 5B) and frontgate mode (Figure 5C). In the backgate mode, the gate of the control transistor is biased at a fixed frontgate voltage while the SiRi is turned ON or OFF by sweeping the backgate voltage (Figure 5B). In the backgate mode, to turn ON the SiRi pixel, sufficient frontgate voltage needs to be applied such that the MOSFET is first turned ON (Figure 5B). In the frontgate mode, the MOSFET is turned ON or OFF by sweeping the gate of the control transistor while the SiRi is biased at a fixed backgate voltage (Figure 5C). In the frontgate mode, to turn ON the SiRi pixel, sufficient backgate voltage needs to be applied such that the SiRi is first turned ON. In the frontgate mode, the SiRi behaves as a resistor but turned ON/OFF using the backgate voltage (Figure 5C).

In the frontgate mode, the gate of the transistor (V_G_) is swept from −2.5 to +2.5 V and an initial bias voltage of −0.1 V for P-metal-oxide-semiconductor (PMOS) (or 0.1 V for N-metal-oxide-semiconductor (NMOS)) was applied to the drain terminal (V_D_) while the source terminal (V_S_) was connected to the ground. The SiRi is turned ON by the application of a constant bias voltage to the backgate (V_BG_). In the backgate mode, an initial bias voltage of −0.1 V for PMOS (or 0.1 V for NMOS) was applied to the drain terminal (V_D_) while the source terminal (V_S_) was connected to the ground. Then, a bias voltage of 0 V was applied to the gate of the control transistor (both NMOS and PMOS), while the backgate was swept from 25 to −10 V for P-type and from 0 to 30 V for N-type SiRi, respectively. In both modes of operation, the fluid gate terminal (V_FG_) was left open and not connected as these measurements were performed prior to bio functionalization. The influence of substrate voltage bias (V_BG_) on standalone N- and P-type transistors was also studied in this work. During the electrical measurements of NMOS transistor, the V_G_ was swept from −2.5 to 2.5 V while keeping the V_S_ at 0 V, V_D_ at 0.1 V and stepping V_BG_ (−15, −10, −5, 0, 5, 10, 15, 20, 25 and 50 V). Similarly, in the case of the PMOS transistor, the V_G_ was swept from −2.5 to 2.5 V while keeping the V_S_ at 0 V, V_D_ at −0.1 V and stepping V_BG_ (−50, −25, −20, −15, −10, −5, 0, 5, 10, 15 V).

Table 2 shows the N- and P-type pixels that were studied in this work. The dimensions of the corresponding control transistor and ribbon module in the respective pixel is also shown. Single N-type SiRi of W = 1 μm and L = 1 μm was connected to N-type control transistor of L = 1 μm and W = 4 μm respectively. Single P-type SiRi of W = 1 μm and L = 1 μm was connected to P-type control transistor of L = 1 μm and W = 4 μm, respectively. The important performance metrics of the SiRi pixels and transistors, namely the subthreshold slope (SS) and threshold voltage (V_TH_), were noted from the transfer characteristics. The linear extrapolation method was employed to extract the V_TH_ of the SiRi pixels and the transistors.

## 4. Results and Discussion

### 4.1. I_D_-V_G_ Transfer Characteristics of N- and P-Type Transistors

**Figure 6:**
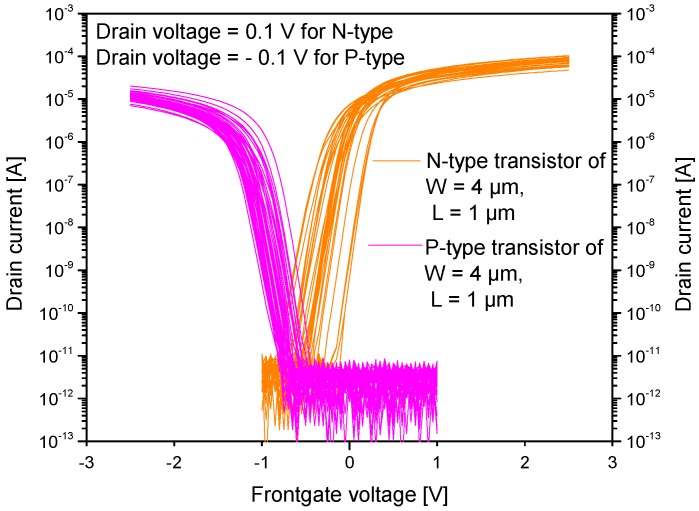
The wafer scale I_D_-V_G_ transfer characteristics of standalone NMOS and PMOS transistors manufactured using the same fabrication process as the SiRi pixels. The backgate voltage is set to 0 V during the measurements.

**Figure 7:**
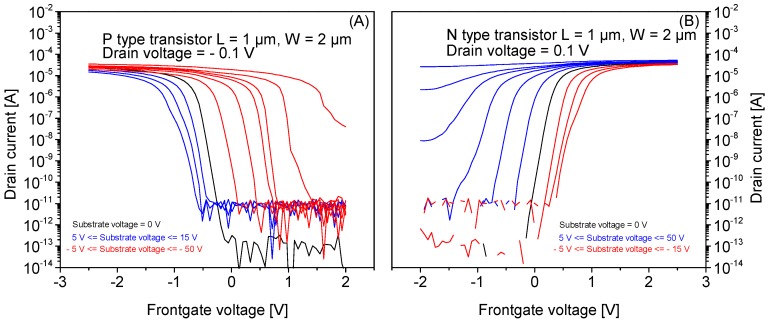
I_D_-V_G_ transfer characteristics of standalone (**A**) P-type and (**B**) N-type control FET at different V_BG_ values. It is clear that application of V_BG_ (>20 V for N-type and <−25 V for P-type) pushes the devices into inversion regime of operation. In addition, the V_TH_ and SS of the devices are impacted by the application of V_BG_.

Figure 6 shows the wafer scale I_D_-V_G_ transfer characteristics (at V_BG_ = 0 V) of standalone NMOS and PMOS transistors manufactured using the same fabrication process as the SiRi pixels. The NMOS and PMOS transistors have a SS of 60–65 mV/dec. The extracted V_TH_ for NMOS is in the range from −0.3 to 0.3 V while the PMOS transistor has V_TH_ in the range of −0.9 to −1.1 V. Figure 7A,B shows the I_D_-V_G_ transfer characteristics of a single PMOS and NMOS device manufactured on SOI wafer at different V_BG_. 

In Figure 7A, the black color I_D_-V_G_ curve of the PMOS is at V_BG_ = 0 V. The positive V_BG_ values cause the I_D_-V_G_ transfer characteristics to shift to the left side (blue color) of the I_D_-V_G_ curve at V_BG_ = 0 V as the back interface is in accumulation. The V_TH_ decreases by ~20–25 mV for 1 V change in V_BG_. The SS is only slightly influenced. The negative V_BG_ values cause the I_D_-V_G_ transfer characteristics to shift more towards the right side (red color) of the I_D_-V_G_ curve at V_BG_ = 0 V as the Si/BOX interface approaches inversion. At −50 V ≥ V_BG_ ≥ −25 V, the frontgate control over the channel is gradually lost causing the increase in SS. As a result, at V_BG_ values < −25 V, the transistor can no longer be turned off. For −25 V ≥ V_BG_ ≥ −5 V, the V_TH_ linearly depends on V_BG_ and changes by ~100 mV for −1 V change in V_BG_.

Similarly, in Figure 7B the black color I_D_-V_G_ curve of the NMOS is at V_BG_ = 0 V. The negative V_BG_ values cause the I_D_-V_G_ transfer characteristics to shift to the right side (red color) of the black curve as the back interface is in accumulation. The V_TH_ linearly increases by ~20−25 mV for 1 V change in V_BG_. The SS is only slightly influenced. Whereas the positive V_BG_ values cause the I_D_-V_G_ transfer characteristics to shift more towards the left side (blue color) of the I_D_-V_G_ curve at V_BG_ = 0 V as the Si/BOX interface approaches inversion. At 50 V ≤ V_BG_ ≤ 15 V, the frontgate control over the channel is gradually lost causing the increase in SS. As a result, at V_BG_ values >25 V, the transistor can no longer be turned off. For 15 V ≤ V_BG_ ≤ 5 V, the V_TH_ linearly depends on V_BG_ and changes by ~100 mV for 1 V change in V_BG_.

Thus, it was found that the backgate voltage strongly influences the SS and V_TH_ of the MOSFETs and directly impacts the transfer characteristics. As a result, at V_BG_ values ≥ 25 V for NMOS (or −25 V for PMOS), the transistor can no longer be turned off. Thus, it is concluded that, for stable operation of the SiRi pixel, it is important to restrict the V_BG_ in voltage range from −10 to 10 V.

### 4.2. Backgate Mode of Operation or I_D_-V_BG_ Transfer Characteristics of N- and P-Type SiRi Pixel Sensors

**Figure 8:**
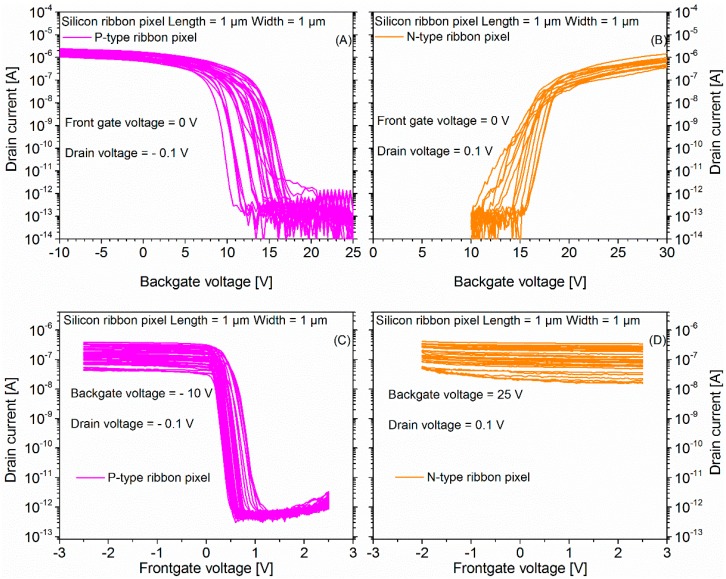
(**A**) I_D_-V_BG_ backgate mode transfer characteristics of P-type SiRi pixel sensor; (**B**) I_D_-V_BG_ backgate mode transfer characteristics of N-type SiRi pixel sensor; (**C**) I_D_-V_G_ frontgate transfer characteristics of P-type SiRi pixel sensor at V_BG_ = −10 V; and (**D**) I_D_-V_G_ frontgate transfer characteristics of N-type SiRi pixel sensor at V_BG_ = 25 V. The N-type pixels cannot turn OFF due to the N-type transistor operating in strong inversion at V_BG_ = 25 V (Figure 7B).

Figure 8A,B shows the wafer scale backgate mode I_D_-V_BG_ transfer characteristics of P- and N-type SiRi pixels (L = 1 μm and W = 1 μm), respectively, with frontgate voltage of the control transistor fixed at 0 V.

In Figure 8B, the N-type sensors conduct a current of value ≥ 1 nA from the source to the drain region when V_BG_ ≥ 15 V while the current values drop to a value of ≤ 1 pA when the V_BG_ is decreased to values lesser than 10 V confirming that the SiRi pixels exhibit characteristics similar to the N-type MOSFETs. The N-type pixel is turned ON as long as the control transistor is biased in the inversion region (V_G_ = 0 V). Similarly, in Figure 8A, the P-type sensors conduct a current of value ≥ 10 nA from the source to the drain region when V_BG_ ≤ 10 V while the current values drop to a value of ≤ 1 pA when the V_BG_ is increased to values greater than 15 V confirming that the SiRi sensors exhibit characteristics similar to the P-type MOSFETs. The P-type pixel is turned ON as long as the control transistor is biased in the inversion region (V_G_ = 0 V). A SS value of 70–80 mV/dec and I_ON_/I_OFF_ ≥ 10^6^ was noted for both N- and P-type sensors.

The V_TH_ of the N-type pixel was found to range from 12.5 to 15.5 V while the V_TH_ of the P-type sensor was found to range from 10 to 15 V. This indicates that the number of charges per unit area in the BOX is 2.2 × 10^12^ cm^−2^. These first observations of high V_TH_ variations also hint at the importance of the backgate interface between c-Si SiRi sensor/BOX layer.

### 4.3. Frontgate Mode of Operation or I_D_-V_G_ Transfer Characteristics of N- and P-Type SiRi Pixel Sensors

Figure 8C,D shows the wafer scale frontgate mode I_D_-V_G_ transfer characteristics of corresponding P- and N-type SiRi pixel devices (L = 1 μm and W = 1 μm), respectively. In Figure 8C,D, the P-type SiRi pixel was biased with V_BG_= −10 V and N-type SiRi pixel device was biased with V_BG_= 25 V, respectively. The I_D_-V_G_ transfer characteristics of N- and P-type SiRi pixel devices is similar to the I_D_-V_G_ transfer characteristics of N- and P-type MOSFET devices, respectively. However, in Figure 8B, the N-type SiRi has high V_TH_ in backgate mode due to the thick BOX layer. In particular, the N-type SiRi (Figure 8B) is in the inversion region of operation at V_BG_ ≥ 20 V. Thus, to turn ON the N-type SiRi pixel in the frontgate mode, the SiRi sensor connected at the output should be biased at V_BG_ ≥ 20 V. When the N-type SiRi pixel sensor was biased with a V_BG_ = 25 V, it was observed that the pixel sensor could not be turned OFF (Figure 8D). This behavior can be understood by referring to the influence of V_BG_ on the I_D_-V_G_ transfer characteristics of N-type transistors that control the SiRi sensor device (Figure 6). It can be noted that N-type standalone transistors have an average V_TH_ of ~0 V and variation of ±0.3 V. Applying a V_BG_ = 25 V to operate the N-type SiRi pixel sensor in the inversion region pushes the I_D_-V_G_ transfer characteristics of the N-type control transistor to even stronger inversion region (Figure 7B) where it can no longer be turned OFF. The V_TH_ of the N-type transistor shifts further to the left side from its ideal operating condition at V_BG_ = 0 V as it moves towards inversion. In the inversion region, the influence of the V_BG_ on the control transistor is stronger than that of the frontgate voltage (V_G_). Since the NMOS transistor controls the N-type SiRi pixel, even the N-type pixels can no longer be turned OFF for V_BG_ ≥ 25 V.

Similarly, the P-type SiRi pixel (Figure 8A) in backgate mode is in the inversion region of operation at V_BG_ ≤ 10 V. Thus, to turn ON the P-type SiRi pixel, the SiRi sensor connected at the output should be biased at V_BG_ ≤ 10 V. When the P-type SiRi pixel sensor was biased with a V_BG_ = −10 V, it was observed that the pixel sensor behaves similar to the P-type MOSFETs (Figure 8C). This behavior is contrary to that of N-type SiRi pixel shown in Figure 8D. This can be understood by referring to the influence of V_BG_ on the I_D_-V_G_ transfer characteristics of P-type transistors that control the SiRi sensor device (Figure 6). In Figure 6, it can be noted that P-type standalone transistors have an average V_TH_ of ~−1.1 V and variation of ±0.1 V. Applying a V_BG_ = −10 V to operate the P-type SiRi pixel sensor in the inversion region pushes the I_D_-V_G_ transfer characteristics of the P-type control transistor to strong inversion region (Figure 7A). The V_TH_ of the P-type pixel sensors is shifted further to the right side from the V_TH_ of the P-type transistors (Figure 8C). Particularly, it can be noted that the V_TH_ of the single P-type pixel sensors is ~0.5 V and variation of ± 0.2 V. However, because of operating the P-type pixels at V_BG_ = −10 V, a relatively low value, the P-type transistor can still be turned OFF. In Figure 8C, the P-type pixels exhibit excellent I_ON_/I_OFF_ ≥ 10^6^, SS of 70–80 mV/dec and V_TH_ of 0.5 V (at V_BG_ = −10 V). However, note that the SiRi connected to the PMOS transistor also behaves as a resistor in series with the PMOS transistor. As a result, the P-type SiRi pixels have a flat saturation characteristic of the ON current. Nonetheless, when operating the P-type pixels for DNA detection or bio-experiments, it is expected to work in the subthreshold region of operation where there is exponential dependence of the drain current with respect to frontgate voltage (V_G_). Hence, the resistance arising due to the SiRi pixel will not influence the detection mechanism. 

Thus, from the results in Figure 8C,D, it can be concluded that the operating voltage of the SiRi pixel sensors directly depends on the V_TH_ of the standalone SiRi sensors connected to the control FET. The quality of the backgate interface between SiRi sensor and BOX plays a key role in determining the operating voltage of the SiRi pixels. If the individual SiRis require larger voltages (>15 V) to turn ON, then the SiRi pixels cannot be turned OFF as the transistors controlling the SiRi sensors are in strong inversion region of operation. Further, the V_TH_ of the individual SiRi sensors depends on the thickness and quality of the BOX layer in the SOI wafer. Since the BOX layer is relatively thick (145 nm), the voltage required to turn ON and turn OFF the standalone SiRi sensors is also very high (25 V for N-type and −10 V for P-type SiRi). In addition, the fixed charges in the BOX layer will significantly impact the V_TH_ of the SiRi pixel sensors. A relatively small change of fixed charges of 3 × 10^11^ cm^−2^ would induce a V_TH_ shift of 2 V due to the BOX thickness of 145 nm. However, as noted shown in Figure 8A,B, the V_TH_ shift is relatively larger (>15 V) indicating that number of fixed charges in the oxide is of the high order ~2.2 × 10^12^ cm^−2^. This issue of V_TH_ variation between devices can be overcome by employing SOI wafers with a thinner and high quality BOX layer or by replacing the BOX layer with a metal backgate.

Finally, as shown in Table 3, it is found that the SiRi pixels operating in the frontgate mode of operation exhibit relatively lower V_TH_ variation in comparison with SiRi pixels operating in the backgate mode. Particularly, the V_TH_ of P-type pixel in the backgate mode operation was found to range from 10 to 15 V while it was reduced to a V_TH_ variation of just 0.3 to 0.7 V in the frontgate mode of operation. The reduction of the V_TH_ in the frontgate mode of operation is due to employing a PMOS transistor to control the SiRi. It can be seen in Figure 6 and Figure 8C that the V_TH_ of the P-type SiRi pixel follows the same V_TH_ variation of its respective control P-type transistor. Similarly, in Table 3, the P-type SiRi pixels operating in the frontgate mode of operation exhibit relatively larger I_ON_ variation (4 × 10^−7^ to 3 × 10^−6^ A) in comparison with SiRi pixels operating in the backgate mode (10^−6^ to 2.5 × 10^−6^ A). We speculate this to arise on account of process variations in the channel resistance of the P-type SiRi that is connected to the P-type control transistor. 

### 4.4. Influence of Backgate Voltage on the SiRi Pixel Sensors in Frontgate Mode of Operation

**Figure 9:**
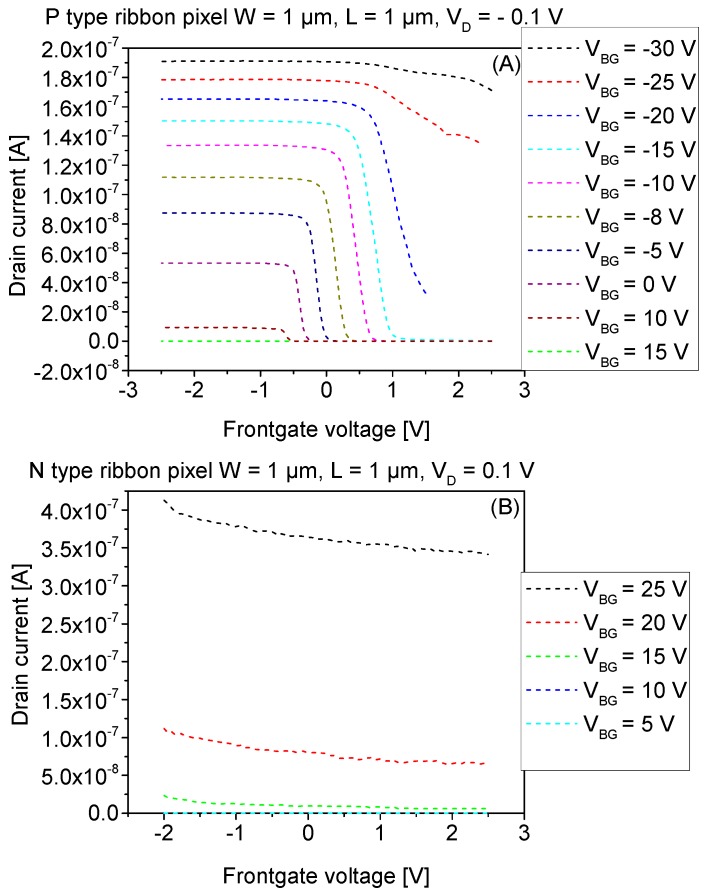
I_D_-V_G_ frontgate mode transfer characteristics of (**A**) P-type and (**B**) N-type SiRi pixel at different V_BG_ values. It is observed that application of V_BG_ causes the modulation of I_D_, V_TH_ and SS of the P-type pixel devices (**A**). The N-type pixel devices (**B**) cannot be turned OFF even by applying 5 V ≤ V_BG_ ≤ 25 V because the standalone N-type SiRi sensor switches ON at V_BG_ ≥ 25 V (Figure 8B). Simultaneously, at V_BG_ ≥ 20 V, the N-type control FET moves to strong inversion (Figure 7B) where it can no longer be turned OFF. As a result, the pixel that is controlled by the N-type FET is also not turned OFF.

The mechanism of tuning the V_TH_ of the SiRi pixel by using the backgate bias can be exploited to match with the operating voltage of the control CMOS circuits. Therefore, we studied the influence of V_BG_ on the N- and P-type pixels in the frontgate mode operation. Figure 9A shows the I_D_-V_G_ frontgate transfer characteristics of a single P-type SiRi pixel device of W = 1 μm and L = 1 μm at different V_BG_. A systematic V_TH_ shift of ~40 mV is observed for 1 V change in V_BG_. The I_D_ of the pixel also increases by ~3–8 nA for 1 V change in V_BG_. Figure 9B shows the I_D_-V_G_ frontgate transfer characteristics of a single N-type SiRi pixel device of W = 1 μm and L = 1 μm at different V_BG_. Since the controlling NMOS transistor is in strong inversion region for 20 V ≤ V_BG_ ≤ 25 V, the N-type SiRi cannot be turned OFF.

In Figure 8A, in the backgate mode, P-type SiRi pixel is operating in the inversion region at V_BG_ = −10 V. Further, in Figure 8C, the P-type pixels can be switched ON by applying a V_BG_= −10 V to the SiRi connected at the output. However, in Figure 9A, i for −1 V ≥ V_BG_ ≥ 0 V, the P-type pixels are in OFF state as the P-type SiRi connected at the output of the pixel is in OFF state. For −4 V ≥ V_BG_ ≥ −2 V, the P-type SiRi at the output of the pixel is in the weak inversion region. Thus, the I_D_ of the P-type pixel is lower (50 nA). At the same time, the application of a V_BG_ = −4 V causes the V_TH_ of the PMOS control transistor to shift towards the right side as the transistor moves towards strong inversion (Figure 7A).

A further increase of the V_BG_ (−20 V ≥ V_BG_ ≥ −6 V), causes the P-type SiRi at the output of the pixel to move towards the strong inversion. As a result, the I_D_ of the P-type pixel also increases 80 nA–0.16 μA (Figure 9A). Concurrently, this increase in V_BG_ (−20 V ≥ V_BG_ ≥ −6 V) causes the V_TH_ of the PMOS control transistor to shift further towards the right side as the transistor moves towards even stronger inversion (Figure 7A). Eventually, at V_BG_ ≤ −20 V, the control PMOS transistor can no longer be turned OFF. Since the P-type SiRi pixel is controlled by the PMOS transistor, even the P-type pixels can no longer be turned OFF for V_BG_ ≤ −20 V. 

Note that, in Figure 8C,D, the frontgate voltage (V_G_) of the N- and P-type pixels is swept from −2.5 to 2.5 V. Further increase of the frontgate voltage than the aforementioned range (−2.5 to 2.5 V) will lead to the dielectric breakdown (4 nm SiO_2_) in the control transistors. Thus, from this dependence of the N- and P-type SiRi pixels (Figure 8D and Figure 9A) on V_BG_, it can be concluded that it is important to restrict the V_TH_ of the N- and P-type SiRi connected at the control FET to low values (5 V ≤ V_BG_ ≤ 10 V for N-type and −5 V ≥ V_BG_ ≥ −10 V for P-type sensors). As long as the V_BG_ value required to turn ON the standalone SiRi sensors is within the operating bounds (−2.5 to 2.5 V) of the transistor controlling them, then the SiRi pixel sensors can be switched ON or OFF. Otherwise, the large V_BG_ value applied to turn ON the SiRi sensors, will push the controlling transistor in the SiRi pixel device, towards stronger inversion region of operation where the influence of backgate voltage (V_BG_) is greater than that of the frontgate (V_G_). As a result, the SiRi pixel devices cease to switch OFF. The thickness and quality of the backgate interface or particularly BOX layer in this study will also strongly influence the V_BG_ required to bias the SiRi sensors. 

## 5. Conclusions

In this paper, the monolithic integration of SiRi sensor with CMOS has been realized using a novel pixel-based LOC architecture. A single pixel sensor comprised of a control MOSFET, an on-chip fluid-gate and a SiRi sensor connected at its output. In particular, single N- and P-type SiRi sensors of dimensions W = 1 μm and L = 1 μm were connected with N- and P-type CMOS transistors of W = 4 μm and L = 1 μm respectively. The top-down method of fabrication was exploited to manufacture the SiRi pixels. Wafer scale integration was exposed on 100 mm SOI substrate using the CMOS industry grade materials and tools. 

Furthermore, we demonstrated the first electrical results of the pixel design for both N- and P-type SiRi pixels in two different modes: (a) the backgate mode; and (b) the frontgate mode. From the I_D_-V_BG_ characteristics in the backgate mode, it is found that the N- and P-type pixels exhibit similar characteristics to that of N- and P-type MOSFETs, respectively, with a SS of 70–80 mV/dec and I_ON_/I_OFF_ ≥ 10^6^. The V_TH_ variation of 12.5–15.5 V for N-type pixel and 10–15 V for P-type pixel was also noted in the backgate mode of operation. Likewise, the I_D_-V_G_ frontgate mode transfer characteristics of N- and P-type SiRi pixel devices were noted to be similar to the I_D_-V_G_ transfer characteristics of N- and P-type MOSFETs, respectively. However, in the frontgate operation, a strong dependence was observed on the quality of the SiRi backgate interface. Since the number of charges in the BOX was high (2.2 × 10^12^ cm^−2^), it caused a large V_TH_ shift in SiRi sensors, which in turn impacted the frontgate mode of operation. It was revealed that individual SiRis require larger voltages (>15 V) to turn ON, and then the SiRi pixels cannot be turned OFF as the transistors controlling the SiRi sensors are in strong inversion region of operation. Particularly, the N-type pixels cannot be turned OFF due to the control NMOS operating in the strong inversion regime. This is because of large V_BG_ application to turn ON the SiRi sensor (V_BG_ ≥ 25 V). Conversely, the P-type SiRi sensors in the frontgate mode do not require large V_BG_ to switch ON. Thus, the P-type pixels exhibit excellent I_ON_/I_OFF_ ≥ 10^6^, SS of 70−80 mV/dec and V_TH_ of 0.5 V (at V_BG_ = −10 V). 

Lastly, we show the influence of different V_BG_ bias on the I_D_-V_G_ frontgate transfer characteristics of a single P-type SiRi pixel device. A systematic V_TH_ shift of ~40 mV is observed for 1 V change in V_BG_. The I_D_ of the pixel also increases by ~3−8 nA for 1 V change in V_BG_. The tuning of the V_TH_ of the SiRi pixel by using the backgate bias will be useful to match with the operating voltage of the control CMOS circuits. Indeed, the novel pixel based design and promising transfer characteristics of SiRi pixels addresses the key area of selectively accessing SiRi sensors using CMOS transistors. Thus, the concept of monolithic integration of SiRi sensors with CMOS technology is successfully demonstrated.

## Figures and Tables

**Table 1:** A summary of some of the most important works employing silicon ribbon (SiRi) sensor for the detection of pH or bio targets. The distinction is made based on fabrication method (top-down (TD) versus bottom-up (BU)), substrate type (Bulk Si versus silicon-on-insulator (SOI)) and type of sensor. Further, the application and complementary-metal-oxide-semiconductor (CMOS) integration feature are also considered.

Reference	Fabrication Method, Substrate, Device Type	CMOS Integration	Application
Zhang et al. [1]	TD, SOI, SiRi	No	Dengue virus (DEN-2)
Lee et al. [2]	TD, SOI, SiRi	No	pH
Park et al. [3]	TD, SOI, SiRi	No	pH
Yoo et al. [4]	TD, SOI, SiRi	No	pH
Nguyen et al. [5]	BU, Bulk, SiNN-FET	No	DNA
Kim et al. [6]	TD, SOI, SiRi	No	PSA cancer marker
Chaing et al. [7]	TD, SOI, SiRi	No	H5N2 virus
Chen et al. [8]	TD, SOI, SiRi	No	pH
Tarasov et al. [9]	TD, SOI, SiRi	No	pH, ions

**Table 2:** Geometrical characteristics of the SiRi pixel devices studied in this work. The thickness of the SiRi sensors is 20 nm. In a N-type pixel, a N-type transistor of L = 1 μm and W = 4 μm is connected to a N-type SiRi sensor of L = 1 μm and W = 1 μm. Similarly, in the P-type pixel, a P-type transistor of L = 1 μm and W = 4 μm is connected to a P-type SiRi sensor of L = 1 μm and W = 1 μm.

Type of Pixel	Type of Transistor	Transistor Dimensions		Type of SiRi	SiRi Dimensions	
		L (μm)	W (μm)		L (μm)	W (μm)
N	N	1	4	N	1	1
P	P	1	4	P	1	1

**Table 3:** A comparative study of the variation of V_TH_ and I_ON_ in the P-type SiRi pixel device in the backgate and frontgate mode of operation.

Electrical Parameter	P-Type SiRi Pixel (Backgate Mode)	P-Type SiRi Pixel (Frontgate Mode)
V_TH_ variation (V)	10 to 15	0.3 to 0.7
I_ON_ variation (A)	1 × 10^−6^ to 2.5 × 10^−6^	4 × 10^−7^ to 3 × 10^−6^
